# 1,3-Dibenz­yloxy-5-(bromo­meth­yl)benzene

**DOI:** 10.1107/S1600536809009672

**Published:** 2009-03-25

**Authors:** Peihua Zhu, Yanfang Zhao, Haiyan Chen, Qingtao Cui, Qin Wei

**Affiliations:** a School of Chemistry and Chemical Engineering, University of Jinan, Jinan 250022, People’s Republic of China

## Abstract

In the title compound, C_21_H_19_BrO_2_, the dihedral angles between the central benzene ring and the two peripheral rings are 50.28 (5) and 69.75 (2)°. The O—CH_2_ bonds lie in the plane of the central ring and adopt a *syn*–*anti* conformation.

## Related literature

For related compounds, see: Pan *et al.* (2005 ▶); Xiao *et al.* (2007 ▶); For the synthesis, see: Hawker & Fréchet (1990 ▶).
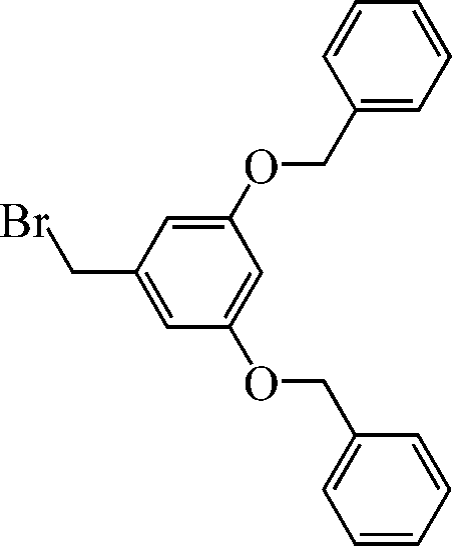


## Experimental

### 

#### Crystal data

C_21_H_19_BrO_2_
*M* *_r_* = 383.27
Triclinic, 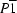

*a* = 4.4449 (17) Å
*b* = 11.982 (5) Å
*c* = 16.726 (6) Å
α = 86.834 (7)°
β = 87.509 (7)°
γ = 86.524 (7)°
*V* = 887.1 (6) Å^3^
*Z* = 2
Mo *K*α radiation
μ = 2.33 mm^−1^
*T* = 298 K
0.20 × 0.15 × 0.10 mm

#### Data collection

Bruker APEXII CCD area-detector diffractometer
Absorption correction: multi-scan (*SADABS*; Sheldrick, 2003 ▶) *T*_min_ = 0.653, *T*_max_ = 0.801
4223 measured reflections
3030 independent reflections
2199 reflections with *I* > 2σ(*I*)
*R*_int_ = 0.023

#### Refinement

*R*[*F* ^2^ > 2σ(*F* ^2^)] = 0.037
*wR*(*F* ^2^) = 0.090
*S* = 1.02
3030 reflections
217 parameters
H-atom parameters constrained
Δρ_max_ = 0.38 e Å^−3^
Δρ_min_ = −0.40 e Å^−3^

### 

Data collection: *APEX2* (Bruker, 2004 ▶); cell refinement: *SAINT-Plus* (Bruker, 2001 ▶); data reduction: *SAINT-Plus*; program(s) used to solve structure: *SHELXL97* (Sheldrick, 2008 ▶); program(s) used to refine structure: *SHELXL97* (Sheldrick, 2008 ▶); molecular graphics: *XP* in *SHELXTL* (Sheldrick, 2008 ▶); software used to prepare material for publication: *XP* in *SHELXTL*.

## Supplementary Material

Crystal structure: contains datablocks global, I. DOI: 10.1107/S1600536809009672/hg2488sup1.cif

Structure factors: contains datablocks I. DOI: 10.1107/S1600536809009672/hg2488Isup2.hkl

Additional supplementary materials:  crystallographic information; 3D view; checkCIF report

## Figures and Tables

**Fig. 1.**
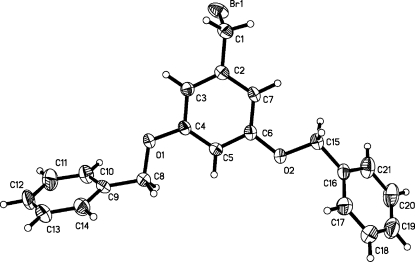
The molecular structure, with atom labels and 25% probability displacement ellipsoids for non-H atoms.

## supplementary crystallographic information

### Comment

The chemistry and physics of dendritic compounds started a decade ago. Today, this science of uniquely shaped molecules, namely, dendrite-shaped molecules, is one of the most exciting topics of contemporary interdisciplinary research. As a part of our structural investigations on dendritic macromolecules, the single-crystal X-ray diffraction study on the title compound was carried out. The compound crystallizes in the triclinic system with a P-1 space group. In the title compound, the O—CH_2_ bonds lie in the plane of the central phenyl ring and adopt a *syn*, *anti* conformation. Comparatively, the O—CH_2_ bonds adopt a *syn*,*syn* conformation in the structure of other analogues reported. (Pan *et al.*2005, Xiao *et al.*2007) The dihedral angles between the central benzene ring and the two peripheral ones are 50.28 (5)°, 69.75 (2)° respectively. Although structure of the title compound is similiar to those reported, the dihedral angles in different compounds are sgnificantly different.(Xiao *et al.*2007)

### Experimental

(3,5-Bis-benzyloxy-phenyl)-methanol (4.89 g, 15 mmol) was prepared by treatment with CBr_4_ (6.23 g, 18.75 mmol) and triphenylphosphine(4.92 g, 18.75 mmol) in THF(85 ml) for 15 min at room temperature.  Conventional workup and purification with silica-gel column chromatography (eluent: chloroform) gave 5.8 g of1,3-Bis-benzyloxy-5-bromomethyl-benzene (65%) as a colorless needles (Hawker & Fréchet, 1990). Single crystals suitable for X-ray study were grown by diffusion method[dichloromethane/*n*-hexane (1:6, V/V)] at room temperature.

### Refinement

All H-atoms bound to carbon were refined using a riding model with distance C—H = 0.93 Å, *U*_iso_ = 1.2*U*_eq_ (C) for aromatic atoms and C—H = 0.97 Å, *U*_iso_ = 1.2*U*_eq_ (C) for methylene atoms.

### Crystal data

**Table:**

C_21_H_19_BrO_2_	*Z* = 2
*M**_r_* = 383.27	*F*(000) = 392
Triclinic, *P*1	*D*_x_ = 1.435 Mg m^−^^3^
Hall symbol: -P 1	Mo *K*α radiation, λ = 0.71073 Å
*a* = 4.4449 (17) Å	Cell parameters from 1804 reflections
*b* = 11.982 (5) Å	θ = 2.4–25.7°
*c* = 16.726 (6) Å	µ = 2.33 mm^−^^1^
α = 86.834 (7)°	*T* = 298 K
β = 87.509 (7)°	Needle, colorless
γ = 86.524 (7)°	0.20 × 0.15 × 0.10 mm
*V* = 887.1 (6) Å^3^	

### Data collection

**Table:**

Bruker APEXII CCD area-detector diffractometer	3030 independent reflections
Radiation source: fine-focus sealed tube	2199 reflections with *I* > 2σ(*I*)
graphite	*R*_int_ = 0.023
Detector resolution: 0 pixels mm^-1^	θ_max_ = 25.0°, θ_min_ = 2.2°
φ and ω scans	*h* = −5→5
Absorption correction: multi-scan (*SADABS*; Sheldrick, 2003)	*k* = −13→14
*T*_min_ = 0.653, *T*_max_ = 0.801	*l* = −19→16
4223 measured reflections	

### Refinement

**Table:**

Refinement on *F*^2^	Primary atom site location: structure-invariant direct methods
Least-squares matrix: full	Secondary atom site location: difference Fourier map
*R*[*F*^2^ > 2σ(*F*^2^)] = 0.037	Hydrogen site location: inferred from neighbouring sites
*wR*(*F*^2^) = 0.090	H-atom parameters constrained
*S* = 1.02	*w* = 1/[σ^2^(*F*_o_^2^) + (0.0439*P*)^2^] where *P* = (*F*_o_^2^ + 2*F*_c_^2^)/3
3030 reflections	(Δ/σ)_max_ = 0.001
217 parameters	Δρ_max_ = 0.38 e Å^−^^3^
0 restraints	Δρ_min_ = −0.40 e Å^−^^3^

### Special details

**Table:**

Geometry. All e.s.d.'s (except the e.s.d. in the dihedral angle between two l.s. planes) are estimated using the full covariance matrix. The cell e.s.d.'s are taken into account individually in the estimation of e.s.d.'s in distances, angles and torsion angles; correlations between e.s.d.'s in cell parameters are only used when they are defined by crystal symmetry. An approximate (isotropic) treatment of cell e.s.d.'s is used for estimating e.s.d.'s involving l.s. planes.
Refinement. Refinement of *F*^2^ against ALL reflections. The weighted *R*-factor *wR* and goodness of fit *S* are based on *F*^2^, conventional *R*-factors *R* are based on *F*, with *F* set to zero for negative *F*^2^. The threshold expression of *F*^2^ > σ(*F*^2^) is used only for calculating *R*-factors(gt) *etc*. and is not relevant to the choice of reflections for refinement. *R*-factors based on *F*^2^ are statistically about twice as large as those based on *F*, and *R*- factors based on ALL data will be even larger.

### Fractional atomic coordinates and isotropic or equivalent isotropic displacement parameters (Å^2^)

**Table:**

	*x*	*y*	*z*	*U*_iso_*/*U*_eq_	
Br1	1.29871 (8)	0.68293 (3)	0.023543 (18)	0.07695 (18)	
O1	0.8518 (4)	0.63235 (14)	0.34038 (10)	0.0550 (5)	
O2	0.9591 (4)	0.27865 (14)	0.22300 (11)	0.0564 (5)	
C19	0.7393 (9)	−0.1122 (3)	0.2047 (3)	0.0876 (12)	
H19	0.6533	−0.1810	0.2129	0.105*	
C5	0.9054 (6)	0.4506 (2)	0.28173 (14)	0.0425 (6)	
H5	0.7738	0.4187	0.3200	0.051*	
C12	0.4165 (10)	0.8523 (4)	0.5579 (2)	0.0898 (12)	
H12	0.3621	0.9099	0.5917	0.108*	
C6	1.0397 (6)	0.3871 (2)	0.22160 (15)	0.0428 (6)	
C2	1.2996 (5)	0.5448 (2)	0.16780 (14)	0.0402 (6)	
C4	0.9692 (6)	0.5614 (2)	0.28420 (14)	0.0422 (6)	
C9	0.5721 (6)	0.6814 (2)	0.45747 (15)	0.0467 (6)	
C16	0.9864 (6)	0.0932 (2)	0.18116 (17)	0.0502 (7)	
C7	1.2350 (6)	0.4338 (2)	0.16442 (14)	0.0425 (6)	
H7	1.3223	0.3908	0.1240	0.051*	
C3	1.1667 (6)	0.6085 (2)	0.22694 (14)	0.0427 (6)	
H3	1.2089	0.6834	0.2287	0.051*	
C10	0.3915 (7)	0.7726 (3)	0.43176 (19)	0.0670 (9)	
H10	0.3194	0.7761	0.3803	0.080*	
C8	0.6577 (6)	0.5895 (2)	0.40320 (15)	0.0493 (7)	
H8A	0.7606	0.5275	0.4326	0.059*	
H8B	0.4789	0.5627	0.3812	0.059*	
C17	0.9854 (8)	0.0362 (3)	0.2548 (2)	0.0673 (9)	
H17	1.0693	0.0677	0.2976	0.081*	
C15	1.1188 (7)	0.2048 (2)	0.16995 (17)	0.0560 (7)	
H15A	1.3311	0.1981	0.1818	0.067*	
H15B	1.1004	0.2336	0.1149	0.067*	
C18	0.8630 (9)	−0.0662 (3)	0.2663 (2)	0.0809 (10)	
H18	0.8654	−0.1037	0.3165	0.097*	
C14	0.6715 (8)	0.6790 (3)	0.53462 (17)	0.0669 (8)	
H14	0.7948	0.6184	0.5531	0.080*	
C13	0.5932 (10)	0.7634 (3)	0.5845 (2)	0.0875 (12)	
H13	0.6611	0.7597	0.6364	0.105*	
C11	0.3172 (9)	0.8579 (3)	0.4810 (2)	0.0894 (11)	
H11	0.1990	0.9199	0.4626	0.107*	
C1	1.5149 (6)	0.5949 (2)	0.10714 (15)	0.0523 (7)	
H1A	1.6408	0.5358	0.0831	0.063*	
H1B	1.6449	0.6426	0.1333	0.063*	
C20	0.7390 (10)	−0.0585 (3)	0.1296 (3)	0.0965 (13)	
H20	0.6564	−0.0909	0.0870	0.116*	
C21	0.8642 (9)	0.0451 (3)	0.1187 (2)	0.0784 (10)	
H21	0.8649	0.0821	0.0683	0.094*	

### Atomic displacement parameters (Å^2^)

**Table:**

	*U*^11^	*U*^22^	*U*^33^	*U*^12^	*U*^13^	*U*^23^
Br1	0.0785 (3)	0.0946 (3)	0.0517 (2)	0.01328 (19)	0.01038 (15)	0.02101 (17)
O1	0.0725 (13)	0.0473 (11)	0.0460 (11)	−0.0111 (10)	0.0159 (9)	−0.0164 (9)
O2	0.0716 (13)	0.0400 (11)	0.0572 (12)	−0.0051 (9)	0.0170 (10)	−0.0124 (9)
C19	0.083 (3)	0.052 (2)	0.129 (4)	−0.0135 (19)	0.010 (2)	−0.024 (2)
C5	0.0473 (16)	0.0435 (15)	0.0366 (14)	−0.0023 (12)	0.0006 (11)	−0.0030 (11)
C12	0.108 (3)	0.085 (3)	0.080 (3)	−0.019 (2)	0.034 (2)	−0.050 (2)
C6	0.0455 (15)	0.0430 (16)	0.0404 (14)	0.0007 (12)	−0.0035 (12)	−0.0077 (12)
C2	0.0348 (14)	0.0499 (16)	0.0364 (13)	−0.0010 (12)	−0.0070 (11)	−0.0034 (12)
C4	0.0452 (15)	0.0466 (16)	0.0353 (13)	0.0001 (12)	−0.0032 (11)	−0.0088 (12)
C9	0.0504 (17)	0.0514 (16)	0.0380 (14)	−0.0055 (13)	0.0068 (12)	−0.0046 (12)
C16	0.0511 (17)	0.0415 (16)	0.0582 (18)	0.0036 (13)	0.0017 (13)	−0.0140 (14)
C7	0.0461 (16)	0.0450 (16)	0.0363 (14)	0.0047 (12)	−0.0003 (11)	−0.0096 (11)
C3	0.0459 (15)	0.0436 (15)	0.0392 (14)	−0.0052 (12)	−0.0025 (12)	−0.0053 (12)
C10	0.071 (2)	0.071 (2)	0.0597 (19)	0.0100 (18)	−0.0068 (16)	−0.0173 (17)
C8	0.0568 (18)	0.0486 (16)	0.0419 (15)	−0.0026 (14)	0.0053 (13)	−0.0030 (12)
C17	0.081 (2)	0.058 (2)	0.065 (2)	−0.0096 (17)	−0.0008 (17)	−0.0114 (16)
C15	0.0654 (19)	0.0506 (17)	0.0520 (17)	−0.0011 (15)	0.0092 (14)	−0.0154 (14)
C18	0.095 (3)	0.060 (2)	0.086 (3)	−0.003 (2)	0.010 (2)	−0.004 (2)
C14	0.090 (2)	0.065 (2)	0.0463 (17)	−0.0059 (17)	−0.0089 (15)	−0.0032 (15)
C13	0.130 (3)	0.091 (3)	0.0449 (19)	−0.025 (3)	0.004 (2)	−0.019 (2)
C11	0.096 (3)	0.069 (2)	0.102 (3)	0.019 (2)	0.007 (2)	−0.029 (2)
C1	0.0479 (17)	0.0649 (18)	0.0443 (15)	−0.0028 (14)	−0.0011 (12)	−0.0059 (14)
C20	0.108 (3)	0.074 (3)	0.115 (4)	−0.012 (2)	−0.026 (3)	−0.045 (3)
C21	0.100 (3)	0.063 (2)	0.075 (2)	0.004 (2)	−0.0199 (19)	−0.0196 (18)

### Geometric parameters (Å, °)

**Table:**

Br1—C1	1.955 (3)	C16—C17	1.374 (4)
O1—C4	1.368 (3)	C16—C15	1.493 (4)
O1—C8	1.424 (3)	C7—H7	0.9300
O2—C6	1.368 (3)	C3—H3	0.9300
O2—C15	1.425 (3)	C10—C11	1.364 (4)
C19—C18	1.348 (5)	C10—H10	0.9300
C19—C20	1.379 (6)	C8—H8A	0.9700
C19—H19	0.9300	C8—H8B	0.9700
C5—C4	1.377 (4)	C17—C18	1.372 (5)
C5—C6	1.387 (3)	C17—H17	0.9300
C5—H5	0.9300	C15—H15A	0.9700
C12—C13	1.352 (5)	C15—H15B	0.9700
C12—C11	1.375 (5)	C18—H18	0.9300
C12—H12	0.9300	C14—C13	1.365 (4)
C6—C7	1.381 (4)	C14—H14	0.9300
C2—C3	1.374 (3)	C13—H13	0.9300
C2—C7	1.383 (4)	C11—H11	0.9300
C2—C1	1.491 (4)	C1—H1A	0.9700
C4—C3	1.391 (4)	C1—H1B	0.9700
C9—C10	1.376 (4)	C20—C21	1.390 (5)
C9—C14	1.380 (4)	C20—H20	0.9300
C9—C8	1.487 (3)	C21—H21	0.9300
C16—C21	1.368 (4)		
			
C4—O1—C8	118.8 (2)	C9—C8—H8A	110.1
C6—O2—C15	118.0 (2)	O1—C8—H8B	110.1
C18—C19—C20	120.7 (4)	C9—C8—H8B	110.1
C18—C19—H19	119.7	H8A—C8—H8B	108.4
C20—C19—H19	119.7	C18—C17—C16	121.3 (3)
C4—C5—C6	119.3 (3)	C18—C17—H17	119.3
C4—C5—H5	120.4	C16—C17—H17	119.3
C6—C5—H5	120.4	O2—C15—C16	108.0 (2)
C13—C12—C11	120.1 (3)	O2—C15—H15A	110.1
C13—C12—H12	119.9	C16—C15—H15A	110.1
C11—C12—H12	119.9	O2—C15—H15B	110.1
O2—C6—C7	123.9 (2)	C16—C15—H15B	110.1
O2—C6—C5	115.2 (2)	H15A—C15—H15B	108.4
C7—C6—C5	120.8 (2)	C19—C18—C17	119.8 (4)
C3—C2—C7	120.1 (2)	C19—C18—H18	120.1
C3—C2—C1	120.2 (2)	C17—C18—H18	120.1
C7—C2—C1	119.7 (2)	C13—C14—C9	121.6 (3)
O1—C4—C5	124.8 (2)	C13—C14—H14	119.2
O1—C4—C3	115.2 (2)	C9—C14—H14	119.2
C5—C4—C3	120.1 (2)	C12—C13—C14	119.6 (3)
C10—C9—C14	117.8 (3)	C12—C13—H13	120.2
C10—C9—C8	120.6 (2)	C14—C13—H13	120.2
C14—C9—C8	121.6 (3)	C10—C11—C12	120.2 (4)
C21—C16—C17	118.5 (3)	C10—C11—H11	119.9
C21—C16—C15	121.0 (3)	C12—C11—H11	119.9
C17—C16—C15	120.5 (3)	C2—C1—Br1	110.84 (18)
C6—C7—C2	119.5 (2)	C2—C1—H1A	109.5
C6—C7—H7	120.3	Br1—C1—H1A	109.5
C2—C7—H7	120.3	C2—C1—H1B	109.5
C2—C3—C4	120.2 (2)	Br1—C1—H1B	109.5
C2—C3—H3	119.9	H1A—C1—H1B	108.1
C4—C3—H3	119.9	C19—C20—C21	118.9 (4)
C11—C10—C9	120.7 (3)	C19—C20—H20	120.5
C11—C10—H10	119.7	C21—C20—H20	120.5
C9—C10—H10	119.7	C16—C21—C20	120.7 (4)
O1—C8—C9	108.1 (2)	C16—C21—H21	119.6
O1—C8—H8A	110.1	C20—C21—H21	119.6
